# Towards electrospray-assisted production of lipid-based synthetic cell assemblies†

**DOI:** 10.1039/d4sm01284d

**Published:** 2025-03-04

**Authors:** Pim Vink, Lawrence W. Honaker, Siddharth Deshpande

**Affiliations:** a Laboratory of Physical Chemistry and Soft Matter, Wageningen University & Research Wageningen The Netherlands siddharth.deshpande@wur.nl

## Abstract

Lipid-based vesicles are widely used, minimalistic model containers for *in vitro* reconstitution of biological systems and engineering synthetic cells. These containers provide a micro-chassis to encapsulate biomolecules and study biochemical interactions. Liposomes are often the most sought-after vesicles owing to their cell-mimicking nature, and numerous bulk and on-chip methods exist for their production. However, exploring the scope of synthetic containers, both in terms of the alternative lipid assemblies as well as newer production methods is useful for expanding the toolbox for synthetic biology. In this paper, we report the development of an electrospray-based technique, which we term “ATPS-templated lipid assemblies *via* electrofusion of SUVs” (ATLAES), to form lipid-based vesicles. Using an aqueous two-phase system (ATPS), free of organic solvents, we demonstrate efficient formation of microscopic vesicles stabilized *via* interfacial lipid assembly. Interestingly, the formed vesicles exhibit a nebulous and disordered, but highly stable coating of lipids, and tend to form interconnected vesicle populations. Remarkably, the lipid assemblies can continue to rearrange and reconfigure over time, leading to spherical vesicles with ultra-thin and smooth lipid coating, suggestive of liposomes. Our work provides a new avenue, in the form of electrospray, to form various lipid-based assemblies using all-aqueous systems and we believe this platform can be further exploited for high-throughput vesicle production and higher-order assemblies.

## Abstract 2

This study reports the development of an electrospray-based technique (ATLAES) using all-aqueous systems to form a rich variety of microscopic vesicles, with nebulous to smooth lipid coatings, providing a new platform for engineering synthetic cells.

## Graphical abstract

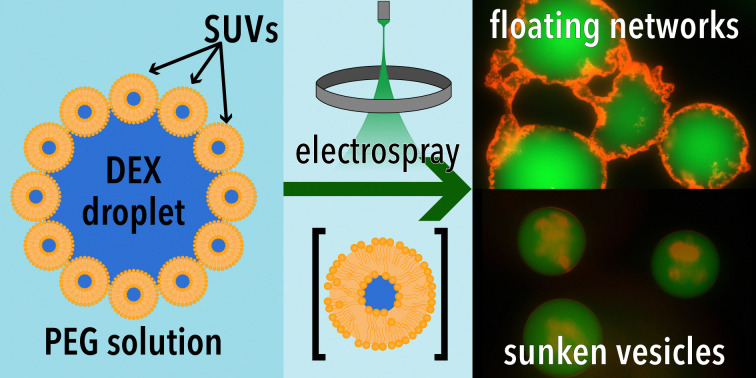

## 1 Introduction

In the quest towards engineering synthetic cells, lipid-based vesicles provide a versatile biocompatible scaffold for macromolecules to be contained in and undergo further interactions and self-organization. Liposomes—spherical aqueous confinements bordered by lipid bilayers—are most often used as a simplified, minimal model for enclosure and encapsulation of biomolecular machinery.^1,2^ However, liposomes are by no means the only possible lipid structures: many other lipid assemblies exist, offering new avenues for the construction and design of lipid-based synthetic cell scaffolds.

Apart from a single, continuous lipid bilayer composing the vesicle, small unilamellar vesicles (SUVs) can also act as an interfacial stabilizer. An aqueous two-phase system (ATPS)—an all-aqueous system composed of two immiscible aqueous phases—can serve as an alternative to complete lipid bilayers when stabilized *via* SUVs, encapsulating biomolecular machinery whilst facilitating rapid exchange of small substrate molecules with their surroundings.^3^ Lipid sponge phases can exhibit a high permeability to the passage of small molecules while still being composed of lamellar structures that can incorporate a variety of membrane proteins.^4^ Interestingly, membrane fragments, originating from lysed red blood cells, were able to stabilize coacervate droplets and form compartments with a relatively disorganized nature whilst retaining permeability and functionality.^5^ Finally, non-lamellar lipid assemblies have garnered interest for drug delivery systems and could become useful scaffolds for synthetic cells.^6^

Liposomes, however, remain the most popular choice and a convenient starting point for developing newer architectures. Majority of the commonly employed liposome production techniques are bulk production methods, such as hydration,^7,8^ electroformation,^9,10^ and inverted emulsion transfer.^11,12^ An increasingly popular option is the generation of double emulsions (water-in-oil-in-water emulsions) through microfluidic methods as starting templates to ultimately form liposomes.^13–17^ These on-chip techniques, on one hand, provide optimal control over vesicle size, lipid composition, and encapsulation.^11,14,18–20^ Both polydimethylsiloxane (PDMS)^21,22^ and glass capillary-based devices^13,23,24^ enable efficient emulsion production.^13,25^ However, these methods need sophisticated setups (such as microfabrication), are generally not suitable for large-scale applications, and require the use of organic solvents to mobilize lipids that can lead to a residual solvent presence in the bilayer.^15,16^ On the other hand, hydration^7,8^ and electroformation^9,10^ techniques are completely free of organic solvents, but offer little process control, especially when encapsulating complex mixtures. In this work, our interest is in exploring and developing novel methods for efficient production of lipid-based emulsions, with the focus being both on good process control and the minimal use of organic solvents.

With this goal in mind, we considered the option to produce emulsions through the use of electrospray atomization, here referred to simply as electrospray. In electrospray, an electric field is applied to a droplet, balancing its interfacial tension against the applied electric field to generate a “Taylor cone”.^26^ The Taylor cone emits a jet that breaks into a spray of small droplets.^27^ These droplets can be collected and further manipulated to produce a variety of colloids and colloidal assemblies.^28–30^ In our previous work, we harnessed electrospray to create self-assembled structures at the interface of ATPS droplets,^31^ using two strongly interacting molecules (a perfluorinated diacid and a pillararene) to ultimately form stable micron-scale vesicles that encapsulated cargo. Since electrospray can work with relatively high throughput,^32^ especially compared to glass capillary- and PDMS-based microfluidics, and can also give us good control over the sizes of the droplets we can produce, we sought to translate this to the production of lipid-based synthetic cell assemblies. The use of electric fields also has considerations with their effects on lipids. Use of electric fields can induce lipid structural rearrangement and promote electroporation^33,34^ and subsequent electrofusion,^35,36^ something we can then make use of when templated by this ATPS interface to promote the fusion of SUVs into larger structures.

In this work, we investigate the formation of lipid-containing structures through electrospray, employing a technique we term “ATPS-templated lipid assemblies *via* electrofusion of SUVs” (ATLAES), where we electrospray dispersions of lipids templated by an aqueous two-phase system of poly(ethylene glycol) (PEG) and dextran (DEX). The PEG–DEX system is a more biocompatible phase-separated system where the two components are aqueous solutions with an inherently ultralow interfacial tension between them,^37–39^ estimated on the order of 10^−1^ mN m^−1^.^40^ We report the formation of uniform-sized DEX droplets that are stabilized by a nebula-like lipid boundary, with the potential to form interconnected structures. Interestingly, some of these chaotic lipid assemblies were found to develop into thin shells over time, completely retaining their internal DEX phase and resembling a liposome. We believe that the ATLAES technique presents us with a possible new route to produce various lipid structures, including liposomes, in a more controlled and all-aqueous manner. This can thus serve to bridge the gap between existing emulsion-based and hydration-based methods, allowing us to produce lipid structures in a greener, more controllable manner.

## 2 Results & discussion

### 2.1 Developing the electrospray set-up for generating ATPS droplets

**Fig. 1:**
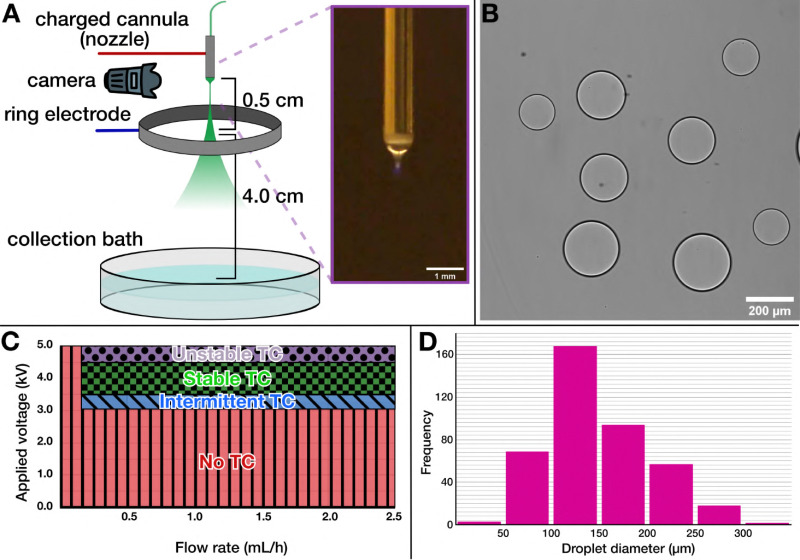
Electrospray set-up for generating ATPS droplets as synthetic cell templates. (A) Schematic of the electrospray set-up showing its core parts: a charged cannula, a charged ring electrode positioned below to generate the electric field, and a collection dish at the bottom to harvest the generated assemblies. Inset shows a micrograph of the Taylor cone formed at the end of the nozzle. (B) A micrograph showing uncoated DEX-in-PEG droplets, demonstrating that DEX solution can be readily electrosprayed into a PEG bath. (C) A electrohydrodynamic phase diagram mapping the effect of applied voltage and the flow rate on Taylor cone formation. As can be seen, a stable Taylor cone forms within a narrow voltage range, and there is a threshold flow rate required for a functioning electrospray. TC: Taylor cone. (D) A sample size distribution of the ATPS droplets obtained by electrospray, corresponding to the production in Fig. 2C. We find a peak size around 100–150 μm (mean ± standard deviation 148 ± 53 μm), but with considerable dispersity at larger droplet sizes.

Like most electrospray set-ups, our apparatus was built in-house, based on the designs used by Song *et al.*,^28^ Vats *et al.*,^41^ and Honaker *et al.*,^31^ as depicted in Fig. 1A and Fig. S1 (ESI†). Our set-up was designed for electrospraying ATPS solutions: we used a blunt-tip cannula as the cathode, through which the solution to be sprayed was flowed using a syringe pump, and an adjustable-height steel ring as the anode. While we found it was possible to electrospray both configurations (DEX into PEG, as seen in Fig. 1(B), or PEG into DEX), we ultimately chose to electrospray DEX into a PEG solution for practical reasons: DEX is readily available with an fluorescein isothiocyanate (FITC) tag to facilitate visualization with fluorescence microscopy; high-concentration DEX solutions are less viscous than PEG solutions of comparable concentration; and the viscosity as a function of solute concentration does not scale as rapidly in DEX^42^ solutions as it does with PEG.^43^ Having a more moderate viscosity results in better control over the Taylor cone, leading to more uniform droplets and longer, more consistent production run times.

We systematically characterized the behavior of the DEX solutions with respect to the applied voltage (*U*) and flow rate (*Q*), as illustrated in Fig. S2 (ESI†), to locate the stable Taylor cone regime for the electrospray amidst many other electrohydrodynamic modes.^44,45^ This characterization process allowed us to identify a reliable, consistent Taylor cone regime across a variety of DEX concentrations. With our set-up, within a voltage range of ∼3.4 kV to 4.2 kV, under a minimal flow of ∼0.2 mL h^−1^ (Fig. 1C), uniformly sized populations of DEX droplets were produced (Fig. 1D). Notably, as the stability of the electrospray was primarily dependent on the solution viscosity (dominated by the DEX), the presence of additional components in the DEX solution did not disrupt the generation of a stable Taylor cone. We also found that solution conductivity was an important parameter to control. A low ion content of the sprayed solution prevents the formation of a stable Taylor cone, whereas a too high salt concentration quickly saturates the solution at the surface of the cone while operating, resulting in precipitates clogging the nozzle and thus blocking the spray. Thus, we aimed for a total salt concentration of ∼150 mM, well within operational limits and benign to processes of lipid assembly.

### 2.2 ATPS-templated lipid assemblies *via* electrofusion of SUVs (ATLAES)

#### 2.2.1 PEGylated lipids drive the assembly of SUV-based membranes at the all-aqueous interface

**Fig. 2:**
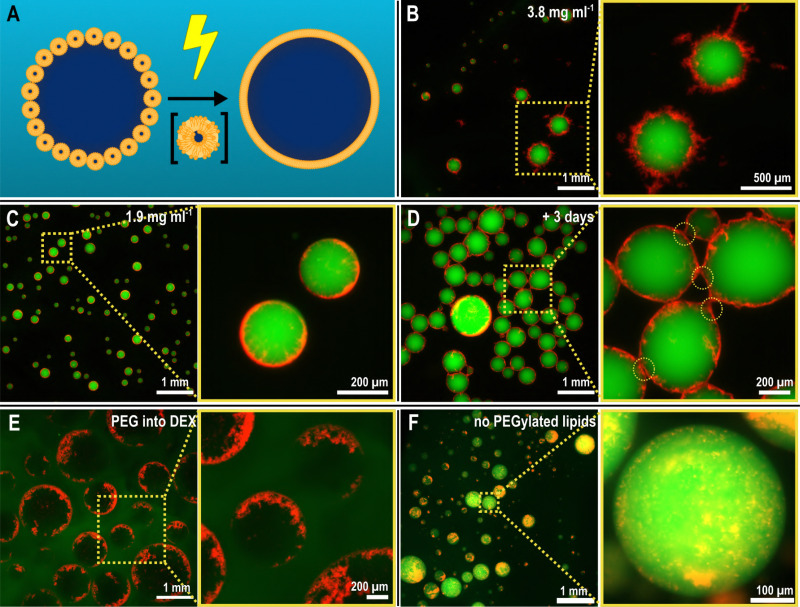
Lipid-coated vesicles are readily formed by electrospraying a dispersion of lipids in a DEX solution into a PEG bath. (A) Schematic of the envisioned electrically-induced fusion of SUVs at the ATPS interface, with an intermediate state of scrambled lipids. (B) Upon electrospraying a DEX solution containing SUVs (prepared with 3.8 mg mL^−1^ lipids) into the PEG bath, the lipids reorganize at the DEX–PEG phase boundary, forming a “nebula”-like structure. (C) Reducing the concentration of electrosprayed SUVs in the DEX phase (prepared with 1.9 mg mL^−1^ lipids) results in relatively smoother shells, but with a similar fuzzy texture. (D) Over time, the lipid-coated DEX droplets form interconnected assemblies *via* the lipid structures at the interface. The connection points show deformed lipid coatings (inset), and minimal droplet coalescence. (E) The localization of lipids at the ATPS interface also occurs in case of an inverted system—that is, spraying the PEG solution into the DEX solution (here, a 4% w/w PEG solution into 10% w/w DEX solution)—albeit with less efficiency. (F) The presence of PEGylated lipids is key to lipid localization: without them, SUVs remain sequestered inside the DEX droplets and do not localize at the PEG–DEX interface. Fluorescence: FITC-DEX (green), Rh-DOPE (red).

After optimizing the electrospray setup and inspired by studies indicating that the fusion of lipid vesicles can be promoted by electrical stimulation,^35,36^ we proceeded with using electrosprayed ATPS systems for creating interfacial lipid assemblies, as depicted in Fig. 2(A). To do so, we first prepared small unilamellar vesicles (SUVs) through extrusion (diameter ≈100 nm; see Methods and Fig. S3 for details,ESI†). We used a mixture of phospholipids (shown in Fig. S4, ESI†) consisting of 1,2-dioleoyl-*sn*-glycero-3-phosophocholine (DOPC) and 1,2-dioleoyl-*sn*-glycero-3-phosphoglycerol (DOPG) (both 36 mol%) as the primary “carrier” lipids with 25 mol% of added 1,2-dioleoyl-*sn*-glycero-3-phospho-l-serine (DOPS), used to promote liposome fusion;^46,47^ 2.9 mol% 1,2-dioleoyl-*sn*-glycero-3-phosphoethanolamine-*N*-[methoxy(poly(ethylene glycol))-2000] (DOPE-mPEG 2000) to promote the localization of SUVs at the ATPS interface; and 0.1 mol% of a fluorophore-tagged DOPE (either rhodamine B, Rh-DOPE, or nitrobenzodiazole, NBD-DOPE) for fluorescence imaging. This SUV dispersion (prepared at 3.8 mg mL^−1^ lipids) was added to a DEX solution which we then electrosprayed into a PEG-containing aqueous outer phase, the results of which are shown in Fig. 2.

Remarkably, this process yielded droplets with a nebulous lipid corona at their interface, depicted in Fig. 2B. Numerous lipid protrusions were visible, extending in- and outwards of the droplets, clearly hinting at a changed lipid organization as a result of the electrospray procedure. Close-up images of the protrusions can be found in the ESI† (Fig. S5). Additionally, these droplets remained afloat after production, at the top of the PEG solution, which was unexpected given that DEX droplets are denser and thus expected to sink in the PEG solution. We attribute this to the low mass of the droplets being unable to counter the interfacial forces that the droplet encounters at the collection of the bath boundary (see ESI,† Tables S1 and S2) and also partly due to the buoyant force from the extensive lipid structures counterbalancing the gravitational pull on the droplets. Reducing the SUV concentration by half in the DEX phase (1.9 mg mL^−1^ lipids) resulted in smoother, less nebulous droplet interfaces (Fig. 2C), clearly showing that the extent of the interfacial assembly can be controlled by simply changing the SUV concentration.

We additionally left these structures to equilibrate after preparation over time. As observed in Fig. 2D, after three days, the droplets remained intact, showing little coalescence and preserving the lipid corona around each of them. The structures, however, began to form agglomerated networks. Some signs of deformation of the lipid nebulae were observable at the interaction points between droplets (Fig. S5, ESI†). This suggests some degree of dynamic nature of the nebulous lipid structures while completely maintaining the stability of the DEX emulsions within the PEG phase.

Concurrent to this, we performed several control experiments to probe what were the essential elements to achieve the observed assembly, detailed in Fig. S6 (ESI†). Absence of an electric field, when dropping the SUV-laden DEX-rich phase into the PEG-rich phase, did not yield any immediate interfacial aggregation, suggesting that indeed the electric field induces rapid lipid organization during the electrospray process. Upon electrospraying the SUV-laden DEX dispersion into a buffer (without PEG, so no ATPS), we merely obtained a solution with lipid aggregates, showing the necessity of the ATPS template for the interfacial assembly. Indeed, spraying the SUV-laden PEG-rich phase into the DEX-rich phase (thus, an inverted system) resulted in interfacial assembly similar to the case of DEX-in-PEG droplets, as seen in Fig. 2E, although less uniformly across the interface. Lastly, we observed the effect of using a PEGylated lipid (PEG2000-DOPE) in the SUVs. The presence of PEGylated lipids was indeed found to be crucial in mobilizing SUVs to the PEG–DEX interface, and this is consistent with previous observations.^3^ In absence of PEGylated lipids, the SUVs simply accumulated inside the DEX-rich phase, resulting in ATPS droplets without any interfacial assembly (as observable in Fig. 2F). We hypothesize that the importance of PEGylation has several aspects. Firstly, PEGylated lipids facilitate the organization of lipid structures, driven by phase separation, at the PEG–DEX interface. However, PEGylated lipids have also been shown to play an important role in the formation and stabilization of lipid bilayer sheets with their open ends shielded by the presence of PEG moieties.^47^ It is hypothesized that Ca^2+^ -induced destabilization of PEGylated SUVs results in the depletion of PEGylated lipids from the contact region between SUVs. Outside the contact region, the PEGylated lipids are concentrated, facilitate opening of the bilayer, and stabilize the open ends of the lamellar structure.^47^ In our work, SUVs were destabilized using a high voltage, which results in pore formation in the lipid bilayer.^34^ Apart from the electric shock promoting vesicle fusion, we hypothesize that the presence of PEGylated lipids can stabilize intermediate bilayer structures that originate when the SUVs are electrically disturbed. These intermediate structures can readily assemble at the ATPS interface in contrast to single lipids, shaping the chaotic nebulae depicted in Fig. 2.

#### 2.2.2 Lipid nebulae can reorganize into smooth continuous boundaries over time

**Fig. 3:**
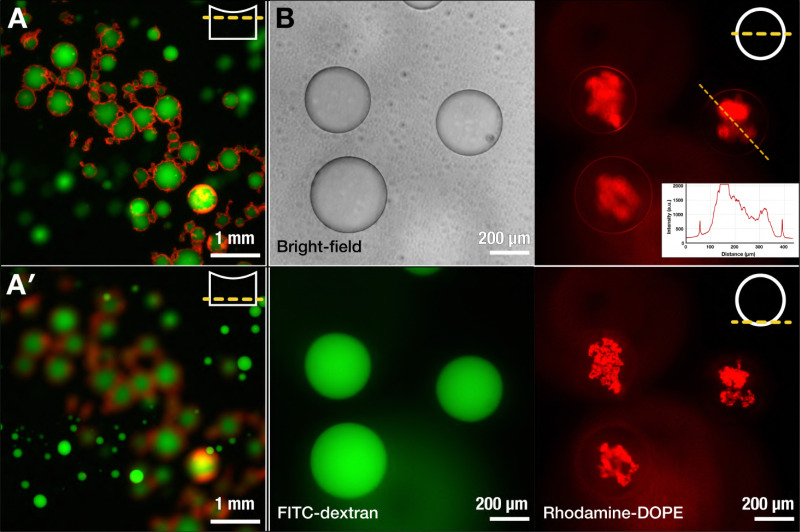
Lipid reorganization over time leads to two distinct populations: floating interconnected droplet assemblies and sunken individual vesicles with ultra-thin lipid shells. (A) and (A′) Upon storing the droplets over at least seven days, we see (A) a population of droplets that remains floating at the top of the dish, showing an interconnected network, and (A′) a number of droplets that have sunk to the bottom and remain as individual vesicles. (B) Visualizing the sunken droplets with bright-field and fluorescence microscopy at the equatorial plane shows distinct, spherical vesicles that are each surrounded by a thin “shell” of lipids. Measuring the lipid fluorescence intensity along the dotted line (inset) shows two clear peaks, corresponding to the vesicle boundary, while a high intensity middle region corresponds to the lipid aggregates located at the DEX droplet pole. Focusing at the bottom of the vesicle makes the nature of lipid reservoirs as aggregated structures more clear. Fluorescence: FITC-DEX (green), Rh-DOPE (red).

While it initially appeared that the lipids remained in a disorganized state at the PEG–DEX interface, we left the produced samples to equilibrate over the course of several days. This led to the accumulation of the lipid-coated ATPS droplets at the center of the dish, likely due to the downward meniscus surrounding each of the droplets becoming pinned at the interface between the droplets, thus causing attractive interactions between particles with similar menisci due to the “Cheerios” effect.^48^ The result was close contact between the lipid nebulae of the droplets that interacted with each other, even resulting in the apparent deformation of droplets, as shown in Fig. 2D. After an incubation period of seven days of leaving the samples undisturbed, the extent of such multi-droplet assemblies was observed to increase both in the number of droplets involved as well as the interfacial contact area between the droplets within the assemblies (Fig. 3A). Interestingly, along with floating multi-droplet assemblies, we also observed numerous individual droplets that had sunk to the bottom of the collection chamber (Fig. 3A′). Likely due to the gradual lipid reorganization at the droplet interface and the constant gravitational pull on the droplets owing to the density difference of the DEX and PEG phases, some of the droplets from the floating interconnected structures, especially those slightly beneath the surface within the cluster, became released, causing them to sink. What was more remarkable, however, was that these sunken droplets showed a very thin and smooth lipid shell as the boundary, encapsulating an internal DEX-rich phase (Fig. 3B and Fig. S5, ESI†). Additionally, they carried a pocket of aggregated lipids at the droplet poles. Thus, it seems likely that a small proportion (less than half) of floating droplets escape the multi-droplet assemblies, settle at the bottom of the container, and their lipids reorganize at the interface to form a continuous, thin boundary at the PEG–DEX interface, with the surplus lipids forming a reservoir. The detachment from the lipid aggregates and the downward migration of the droplets likely promoted the lipid reorganization at the interface, ultimately resulting in a smooth, thin interfacial layer. Several repeats of the experiment, each under slightly differing experimental conditions, showed the same evolution of the structures, beginning with the appearance of a nebulous corona around the floating droplets to the development of a thin lipid layer surrounding the sunken droplets.

Notably, the rate of sinking of droplets played a significant role in facilitating lipid reorganization. By using a low-concentration PEG solution to collect the electrosprayed droplets (≤7% w/w), the droplets sunk too quickly to allow for the gradual reorganization of lipids and the consequent formation of a layered structure. With a 10% w/w PEG solution used for collection, the droplets sunk slowly enough to allow the gradual reorganization of lipids at the interface. On the other hand, using a 15% w/w solution of PEG resulted in no interfacial organization of the lipids to begin with. This may be due to effects arising from the higher viscosity of the more concentrated PEG solution imposing a higher viscous drag. Because of the affinity of the PEGylated headgroups for the PEG phase, we expect some degree of protrusion of the PEGylated chains extending into the aqueous PEG phase. A consequence of this, however, would be that the lipid structures at the interface cannot effectively move along the surface or between the droplets owing to the greater drag imposed by the higher viscosity of the more concentrated PEG solution.

#### 2.2.3 The nebulous structures are solid-like while the smooth lipid boundaries are more fluid and dynamic

**Fig. 4:**
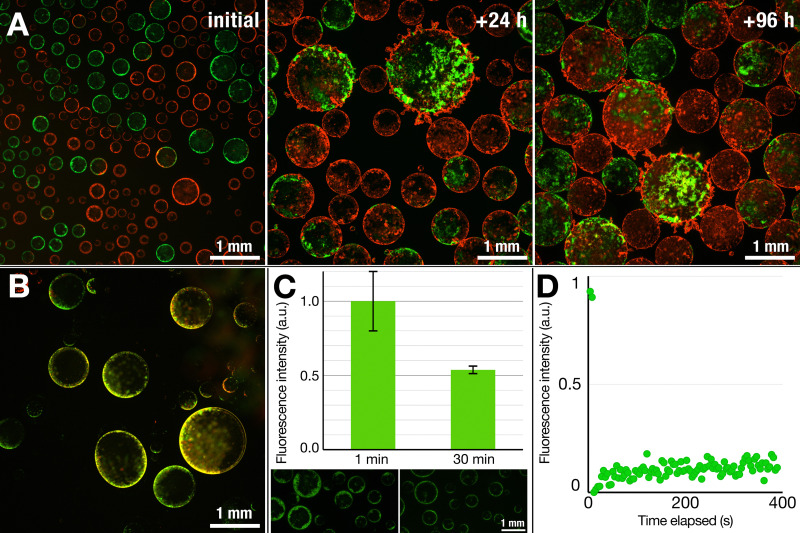
Nebulous lipid assemblies are static and solid-like, while the thin lipid shells are more dynamic. (A) Mixing two vesicle populations containing two different fluorescently labeled lipids (Green: NBD-PE, red: Rhodamine-PE) shows distinct populations at the start. Over time, however, the structures fuse and coalesce, resulting in mixed structures but still showing segregated patches of the NBD- and Rhod-PE-tagged lipids within the same vesicles, even after four days. This suggests that the lipid structures are fluid to only a limited degree. (B) The droplets that detach from the surface and sink, however, show appreciable reorganization of the lipids across the interface, as highlighted by the overlap of the two fluorescent signals (yellow) rather than showing distinct red and green regions. Overall, these results suggest that the structures that float are more solid-like while the structures that sink are more dynamic in nature. (C) A dithionite assay on lipid structures containing NBD-PE lipids shows a reduction of fluorescent intensity by half (0.54 ± 0.03; *n* = 14 different vesicles), a behavior commonly associated with the presence of lipid bilayers. (D) FRAP measurements of the floating structures do not show a fluorescent recovery over time, again suggesting that the lipid structures are not fluid, but rather static structures. The graph represents an average of two measurements.

A natural question we wanted to address next was whether we have smectic-like structured and dynamic interface (similar to a lipid bilayer) or a more solid and static, “jammed” structure present at the ATPS interface. To test this, we mixed samples containing lipid structures prepared with two different fluorophores: the rhodamine-tagged lipids we used earlier and nitrobenzoxadiazole (NBD)-tagged lipids, each with distinct excitation wavelengths. Electrosprayed droplet populations resulting from these two distinctly fluorescent lipid nebulae were then mixed and observed over time. Initially existing as two separate populations of droplets, coalescence of droplets occurred over time, yielding droplets with mixed populations of lipids at their interface. In the case of liquid-like behavior, one would expect the lipids to rearrange at a molecular scale and form a homogeneous mixture over time; however, we did not observe a homogeneous fluorescence but rather binary patches resulting from coalescence of droplets, forming heterogeneous structures which remained even after 96 h, as shown in Fig. 4A.

On the other hand, the structures that detached and sank from the surface were much more prone to reorganization. The test involving two lipid populations showed that, in contrast to the case of their nebulous counterparts, the lipids at the interface of the sunken droplets became mixed (Fig. 4B). Except for a few discrete fluorescent lipid patches, the majority of the thin shell was a mixture of the two lipid compositions, as inferred from the merged fluorescence.

In another test, we performed a dithionite assay using the NBD-tagged lipids, a common test to probe for the formation of lipid bilayers.^49,50^ The addition of dithionite (here, in the form of sodium dithionite) will quench the fluorescence of the NBD fluorophore by converting it to a non-fluorescing aminobenzoxidiazole group: reduction of the fluorescent intensity by half upon addition of dithionite would thus suggest the presence of a bilayer. We prepared dispersions of SUVs containing NBD-labeled DOPE, added the SUV dispersion to a DEX solution, and electrosprayed in the same manner as in previous experiments. To the dispersion of nebulous dextran droplets, we added a dithionite solution to quench the NBD fluorophore. Within 30 min, we observed a roughly 50% decrease in the fluorescence intensity (0.54 ± 0.03) (Fig. 4B), indicating that the lipid nebulae are primarily composed of lipid bilayers. While it is possible that the observed reduction results from completely intact SUVs stabilizing the interface, the electric field stimulation that the SUVs experience and the obtained optical images do suggest that some level of lipid reorganization is likely taking place while retaining a bilayer structure.

To gain further understanding of the lipid nebulae, we performed fluorescence recovery after photobleaching (FRAP) measurements to probe whether they were more solid-like or liquid-like. We observed no fluorescence recovery after 6 min, thus ruling out the possibility of a fluid and continuous lipid bilayer, which would reorganize rather quickly.^51^ This thus indicates a rather solid, aggregated state of lipids that compose the nebulous structures. As was also noticed from the robustness of these structures against the shear forces generated during pipetting, these solid-like nebulae provide protection against mechanical perturbation. The sunken structures with smooth lipid shells, however, proved much more fragile, not allowing us to transfer them between solutions for dithionite or FRAP studies. Thus, while optical observations point towards the presence of a continuous bilayer structure, further investigation is necessary to clarify the exact nature of the thin-shelled lipid vesicles.

## 3 Conclusions

In this work, we have presented ATLAES, an electrospray-based technique to form lipid-bound synthetic vesicles without the use of any organic solvents. Starting with ATPS droplets and promoting lipid assembly at their interface through electrofusion of SUVs, we are able to produce microcontainers stabilized *via* unusual lipid nebulae as well as ultra-thin smooth lipid shells over time, reminiscent of liposomes. Our approach offers new routes for making micro-confinements that can be useful for making new varieties of synthetic cells. The involvement of other electrically active systems, such as polyelectrolytes, may greatly enhance the effectiveness and scope of the presented system. The use of a bulk electrospray method also brings advantages for the eventual scaling up of the production process and more cost-efficient production methods.

The method makes use of the electrical susceptibility of SUVs to reorganize into shell-like structures at the ATPS interface. We hypothesize that, during the electrospraying process, an array of lipid intermediates, such as open bilayers, micelles, and aggregates form that are then consequently stabilized due to the presence of PEGylated lipids. Upon landing in the PEG phase, these intermediates localize at the ATPS interface, facilitated by the presence of PEGylated lipids forming solid-like, nebulous structures. Over time, these nebulae have a chance of developing into interconnected assemblies as well as into smoother, ultra-thin shells upon detaching from the interconnected population.

Further investigation can span multiple directions. For example, the variety of lipids that can be used in this method can be further explored. In this work, we used various lamellar lipids, but electrospray offers the possibility for non-bilayer-forming lipids to be explored for the creation of new lipid assemblies. The encapsulating properties of the presented lipid structures should also be explored further, in order to determine the cut-off molecular size for successful sequestration of biomolecules inside the vesicles. Finally, there is room for further optimization of the ATLAES technique, from the fine-tuning of the PEG–DEX system, to the lipid compositions that are sprayed and the ionic strengths of the solutions used. The latter will likely play a huge role in fusion/aggregation of lipid vesicles, especially when including divalent ions, but may also impact the entire process. In conclusion, ATLAES paves yet another avenue in broadening the scope of producing biomimetic microsystems.

## 4 Materials & methods

### 4.1 Materials

Ultrapure MilliQ water (resistivity 18.2 MΩ cm, total organic carbon ≤ 5 ppb, Merck Millipore) was used to prepare aqueous solutions. The lipids 1,2-dioleoyl-*sn*-glycero-3-phosophocholine (DOPC); 1,2-dioleoyl-*sn*-glycero-3-phospho-l-serine (DOPS); 1,2-dioleoyl-*sn*-glycero-3-phosphoglycerol (DOPG); 1,2-dioleoyl-*sn*-glycero-3-phosphoethanolamine-*N*-[methoxy(poly(ethylene glycol))-2000] (DOPE-mPEG 2000); 1,2-dioleoyl-*sn*-glycero-3-phosphoethanolamine-*N*-(lissamine rhodamine B sulfonyl) (Rh-DOPE); and 1,2-dioleoyl-*sn*-glycero-3-phosphoethanolamine-*N*-(7-nitro-2-1,3-benzoxadiazol-4-yl) (NBD-DOPE), all dissolved in chloroform, were sourced from Avanti Polar Lipids. Dextran (DEX) from *Leuconostoc supp.* (*M*_w_ 150 kDa), fluorescein isothiocyanate-tagged dextran (FITC-dextran, *M*_w_ 20 kDa), poly(ethylene glycol) (PEG, *M*_w_ 35 kDa), sodium dithionite (EMPLURA, ≥ 85%), and phosphate buffered saline (PBS) powder were all sourced from Merck/Sigma Aldrich. All materials were used as supplied without further purification. The physical parameters (*e.g.*, viscosity, density, and interfacial tension) of the ATPS solutions can be found in the ESI† (Table S1).

### 4.2 Solution preparation

#### 4.2.1 Preparation of lipids for ATLAES

We prepared SUVs by lipid film rehydration and subsequent extrusion. Lipids, dissolved in chloroform, were mixed in the desired molar ratio (0.1% fluorescent lipid, 2.9% PEGylated lipid, 97% carrier lipids). The carrier lipid composition was typically divided into 25% DOPS, 36% DOPG, and 36% DOPC, molar ratio. Chloroform was removed under a gentle stream of nitrogen and subsequently desiccated *in vacuo*. The resulting lipid film was rehydrated for 72 h at room temperature in PBS (pH 7.4) to reach a total lipid concentration of 7.5 mg mL^−1^. After rehydration, the lipid suspension was extruded 13 times with an Avanti Polar Lipids mini extruder through a polycarbonate membrane with pore size 100 nm. Dynamic light scattering was performed on the extruded vesicles for size characterization (see Fig. S3, ESI†).

#### 4.2.2 ATPS solutions

A PEG–DEX ATPS was prepared at a 10/10 w/w% composition in PBS (pH 7.4) and left to equilibrate overnight. The PEG-rich phase was gently pipetted from the top and DEX-rich phase was collected by perforating the bottom of the tube and draining it. The DEX-rich phase was labelled with typically 4 wt% of FITC-DEX for contrast and fluorescent imaging. The SUV-laden DEX solutions were typically electrosprayed at a flow rate of 1 mL h^−1^, voltage of 3.5 kV, and collected in a PEG-rich phase, unless stated otherwise.

### 4.3 Electrospray set-up

We built an electrospray set-up as used by Honaker *et al.*,^31^ itself adapted from the set-up developed by Song *et al.*^28^ and Vats *et al.*^41^ A DC high voltage supply generated an electric potential over the cathode, attached to a blunt needle (21G blunt Sterican cannula, sourced from Fisher Scientific B.V.), and an anode attached to a stainless steel ring (height 1 cm, diameter 5 cm). Liquid was fed to the needle using a syringe pump equipped with a 5 mL glass syringe. Tubing and needles were connected using Elveflow microfluidics connectors and Luer locks. The spray nozzle was imaged using a ThorLabs Zelux 1.6 MP Color CMOS Camera with a Navitar zoom lens. Electrosprayed droplets were collected, unless stated otherwise, in cell culture dishes with appropriate collecting solution and typically placed on a rotating stage. The rotating stage (∼0.5–2.0 rad s^−1^) was designed, 3D-printed, and built in-house. Once collected, droplets were imaged with a Zeiss Axio Observer inverted optical microscope equipped with a Colibri light source, a Prime BSI Express camera, and with 2.5× and 10× objectives.

### 4.4 Bilayer tests

#### 4.4.1 Dithionite assay

We electrosprayed droplets using NBD-DOPE as the fluorescently tagged lipid. Once collected, we added an aqueous 1 M sodium dithionite solution to the collection bath in a 1 : 20 ratio relative to the total volume and collected fluorescence micrographs at different times up to 30 min, maintaining the camera acquisition parameters constant throughout the image collection. We then used ImageJ to quantify the fluorescent intensity of the droplets at the start (1 min) and at the end (30 min). Droplets were selected by defining a circular region of interest around them and their intensity was measured at both the time points. Mean fluorescence intensity and standard deviation for each time point were calculated accordingly.

#### 4.4.2 Fluorescence recovery after photobleaching

We electrosprayed droplets, labelled with Rh-DOPE, as previously described. The vesicles were gently pipetted onto microscopy slides and imaged using a Zeiss LSM510 confocal laser scanning microscope. After photobleaching, measurements were taken for seven minutes. The intensity of the bleached area was normalized, relative to an unbleached area, using the equation:1
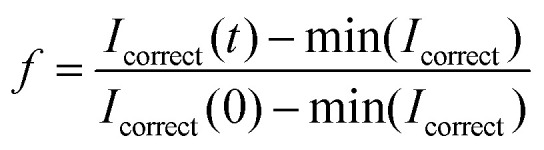
where *I*_correct_ = *C*(*t*)·*I*(*t*), 
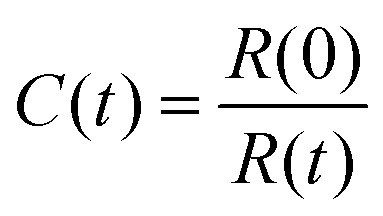
; *R*(*t*) is the fluorescence intensity of the unbleached area for reference at time *t*, *I*(*t*) is the raw fluorescence intensity of the bleached area at time *t*, and min(*I*_correct_) is the minimum value of *I*_correct_, which is obtained right after bleaching the sample. The FRAP experiment was performed in duplicate on two individual vesicles.

## Author contributions

Conceptualization—P. V., L. W. H, S. D.; data curation—P. V.; formal analysis—P. V., L. W. H, S. D.; funding acquisition—S. D.; investigation—P. V.; methodology—P. V., L. W. H., S. D.; project administration—L. W. H, S. D.; resources—L. W. H., S. D.; software—P. V.; supervision—L. W. H., S. D.; validation—P. V., L. W. H.; visualization—P. V., L. W. H., S. D.; writing – original draft—P. V., L. W. H.; writing – review & editing—P. V., L. W. H., S. D. All authors have read and have given their consent to the final version of the publication.

## Data availability

The data supporting this article have been included as part of the ESI.† Any additional data are available upon from the authors upon reasonable request.

## Conflicts of interest

There are no conflicts to declare.

## Supplementary Material

SM-021-D4SM01284D-s001

SM-021-D4SM01284D-s002

## References

[cit1] Ganar K. A., Honaker L. W., Deshpande S. (2021). Curr. Opin. Colloid Interface Sci..

[cit2] Deshpande S., Dekker C. (2021). Curr. Opin. Colloid Interface Sci..

[cit3] Rowland A. T., Keating C. D. (2021). Soft Matter.

[cit4] Bhattacharya A., Niederholtmeyer H., Podolsky K. A., Bhattacharya R., Song J.-J., Brea R. J., Tsai C.-H., Sinha S. K., Devaraj N. K. (2020). Proc. Natl. Acad. Sci. U. S. A..

[cit5] Liu S., Zhang Y., Li M., Xiong L., Zhang Z., Yang X., He X., Wang K., Liu J., Mann S. (2020). Nat. Chem..

[cit6] Zhai J., Fong C., Tran N., Drummond C. J. (2019). ACS Nano.

[cit7] Rideau E., Wurm F. R., Landfester K. (2019). Adv. Biosyst..

[cit8] Cakmak F. P., Marianelli A. M., Keating C. D. (2021). Langmuir.

[cit9] Angelova M. I., Dimitrov D. S. (1986). Faraday Discuss. Chem. Soc..

[cit10] Uzun H. D., Tiris Z., Czarnetzki M., López-Marqués R. L., Günther Pomorski T. (2024). Eur. Phys. J.:Spec. Top..

[cit11] Moga A., Yandrapalli N., Dimova R., Robinson T. (2019). ChemBioChem.

[cit12] Pautot S., Frisken B. J., Weitz D. (2003). Langmuir.

[cit13] Utada A. S., Lorenceau E., Link D. R., Kaplan P. D., Stone H. A., Weitz D. A. (2005). Science.

[cit14] Deshpande S., Caspi Y., Meijering A. E. C., Dekker C. (2016). Nat. Commun..

[cit15] Deshpande S., Dekker C. (2018). Nat. Protoc..

[cit16] Chen C., Ganar K. A., Deshpande S. (2023). J. Visualized Exp..

[cit17] Chen C., Ganar K. A., de Haas R. J., Jarnot N., Hogeveen E., de Vries R., Deshpande S. (2024). Commun. Chem..

[cit18] Van de Cauter L., Fanalista F., van Buren L., Franceschi N. D., Godino E., Bouw S., Danelon C., Dekker C., Koenderink G. H., Ganzinger K. A. (2021). ACS Synth. Biol..

[cit19] Van de Cauter L., van Buren L., Koenderink G. H., Ganzinger K. A. (2023). Small Methods.

[cit20] de Haas R. J., Ganar K. A., Deshpande S., de Vries R. (2023). ACS Appl. Mater. Interfaces.

[cit21] Yandrapalli N., Petit J., Bäumchen O., Robinson T. (2021). Commun. Chem..

[cit22] Trantidou T., Elani Y., Parsons E., Ces O. (2017). Microsyst. Nanoeng..

[cit23] Shah R. K., Shum H. C., Rowat A. C., Lee D., Agresti J. J., Utada A. S., Chu L.-Y., Kim J.-W., Fernandez-Nieves A., Martinez C. J., Weitz D. A. (2008). Mater. Today.

[cit24] Jampani V. S. R., Volpe R. H., Reguengo de Sousa K., Ferreira Machado J., Yakacki C. M., Lagerwall J. P. F. (2019). Sci. Adv..

[cit25] Seemann R., Brinkmann M., Pfohl T., Herminghaus S. (2011). Rep. Prog. Phys..

[cit26] Taylor G. (1964). Proc. R. Soc. London, Ser. A.

[cit27] Zeleny J. (1914). Phys. Rev..

[cit28] Song Y., Chan Y. K., Ma Q., Liu Z., Shum H. C. (2015). ACS Appl. Mater. Interfaces.

[cit29] Maruyama T., Fukui Y., Tsuchiya E., Fujii A. (2012). RSC Adv..

[cit30] Vilabril S., Nadine S., Neves C. M. S. S., Correia C. R., Freire M. G., Coutinho J. A. P., Oliveira M. B., Mano J. F. (2021). Adv. Healthcare Mater..

[cit31] Honaker L. W., Gao T., de Graaf K. R., Bogaardt T. V. M., Vink P., Stürzer T., Kociok-Köhn G., Zuilhof H., Miloserdov F. M., Deshpande S. (2024). Adv. Sci..

[cit32] Gross J. H. , Electrospray Ionization, Springer International Publishing, Cham, 2017, pp. 721–778

[cit33] Rems L., Tang X., Zhao F., Pérez-Conesa S., Testa I., Delemotte L. (2022). eLife.

[cit34] Kotnik T., Rems L., Tarek M., Miklavčič D. (2019). Annu. Rev. Biophys..

[cit35] Rems L., Ušaj M., Kandušer M., Reberšek M., Miklavčič D., Pucihar G. (2013). Sci. Rep..

[cit36] Tresset G., Takeuchi S. (2004). Biomed. Microdevices.

[cit37] Stenekes R. J., Franssen O., van Bommel E. M., Crommelin D. J., Hennink W. E. (1999). Int. J. Pharm..

[cit38] Fick C., Khan Z., Srivastava S. (2023). Mater. Adv..

[cit39] Wu Y.-T., Zhu Z.-Q. (1999). Chem. Eng. Sci..

[cit40] Forciniti D., Hall C. K., Kula M. R. (1990). J. Biotechnol..

[cit41] Vats S., Honaker L. W., Basoli F., Lagerwall J. P. F. (2022). Liq. Cryst..

[cit42] González C., Rubio R., Zenteno-Savin T. (2003). Am. J. Physiol.: Heart Circ. Physiol..

[cit43] Gonzalez-Tello P., Camacho F., Blazquez G. (1994). J. Chem. Eng. Data.

[cit44] Lee A., Jin H., Dang H.-W., Choi K.-H., Ahn K. H. (2013). Langmuir.

[cit45] Kim J. Y., Lee S. J., Baik G. Y., Hong J. G. (2021). ACS Omega.

[cit46] Schlegel R. A., Williamson P. (2001). Cell Death Differ..

[cit47] Grad P., Edwards K., Gedda L., Hernández V. A. (2024). Biochim. Biophys. Acta, Biomembr..

[cit48] Vella D., Mahadevan L. (2005). Am. J. Phys..

[cit49] Angeletti C., Nichols J. W. (1998). Biochemistry.

[cit50] Schaich M., Cama J., Nahas K. A., Sobota D., Sleath H., Jahnke K., Deshpande S., Dekker C., Keyser U. F. (2019). Mol. Pharmaceutics.

[cit51] Pincet F., Adrien V., Yang R., Delacotte J., Rothman J. E., Urbach W., Tareste D. (2016). PLoS One.

