# Supplementary material for: Towards electrospray-assisted production of lipid-based synthetic cell assemblies
Source: Soft Matter. 2025 Mar 4;21(16):2977–85. doi: 10.1039/d4sm01284d (PMC11878373; doi:10.1039/d4sm01284d)
Supplement: SM-021-D4SM01284D-s002 [file SM-021-D4SM01284D-s002.pdf]

Supplementary Information (SI) for Soft Matter.
This journal is © The Royal Society of Chemistry 2025

Supporting Information for "Towards electrospray-assisted production of lipid-based synthetic cell assemblies"

Pim Vink[a], Lawrence W. Honaker[a,b], and Siddharth Deshpande[a,*]

[a] *Laboratory of Physical Chemistry and Soft Matter, Wageningen University & Research, The Netherlands*
[b] *Current affiliation: Experimental Soft Matter Physics Group, University of Luxembourg, Grand Duchy of Luxembourg*

Contents

A Electrospray set-up and calibration procedure **S2**

B Dynamic Light Scattering Characterization of Vesicles **S4**

C Materials **S5**

D Parameters and dimensionless flow numbers associated with our system **S6**

E Additional Images **S7**

A   Electrospray set-up and calibration procedure

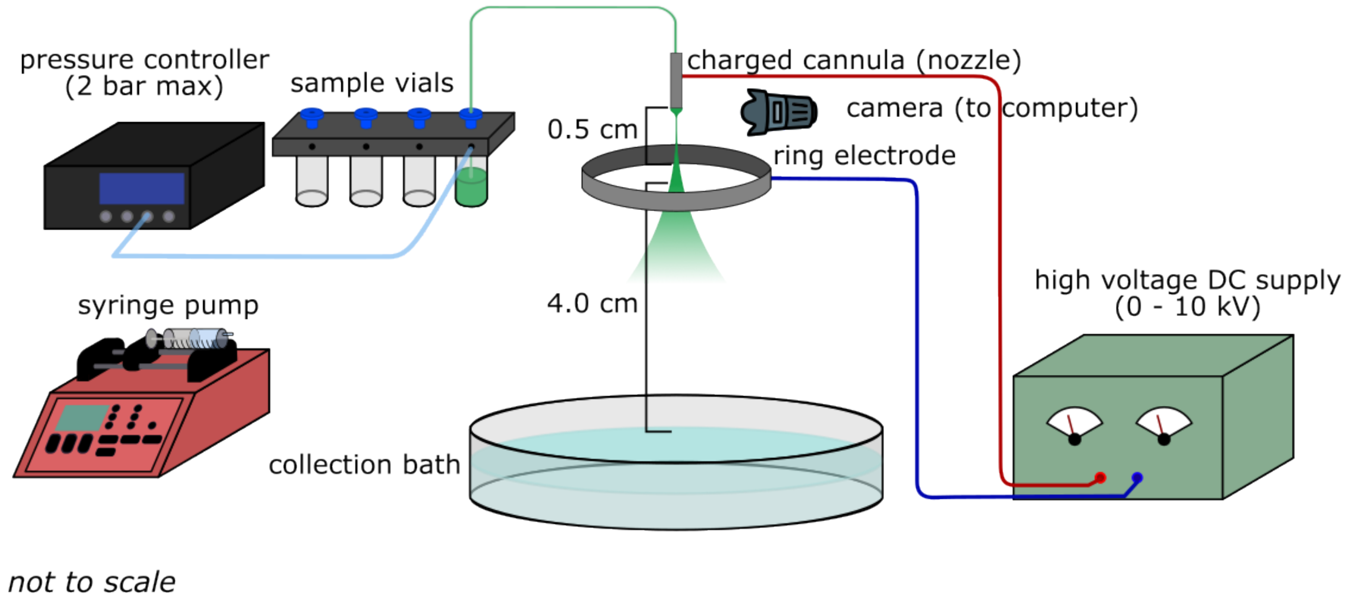

Fig. S1 A detailed schematic of the electrospray set-up.

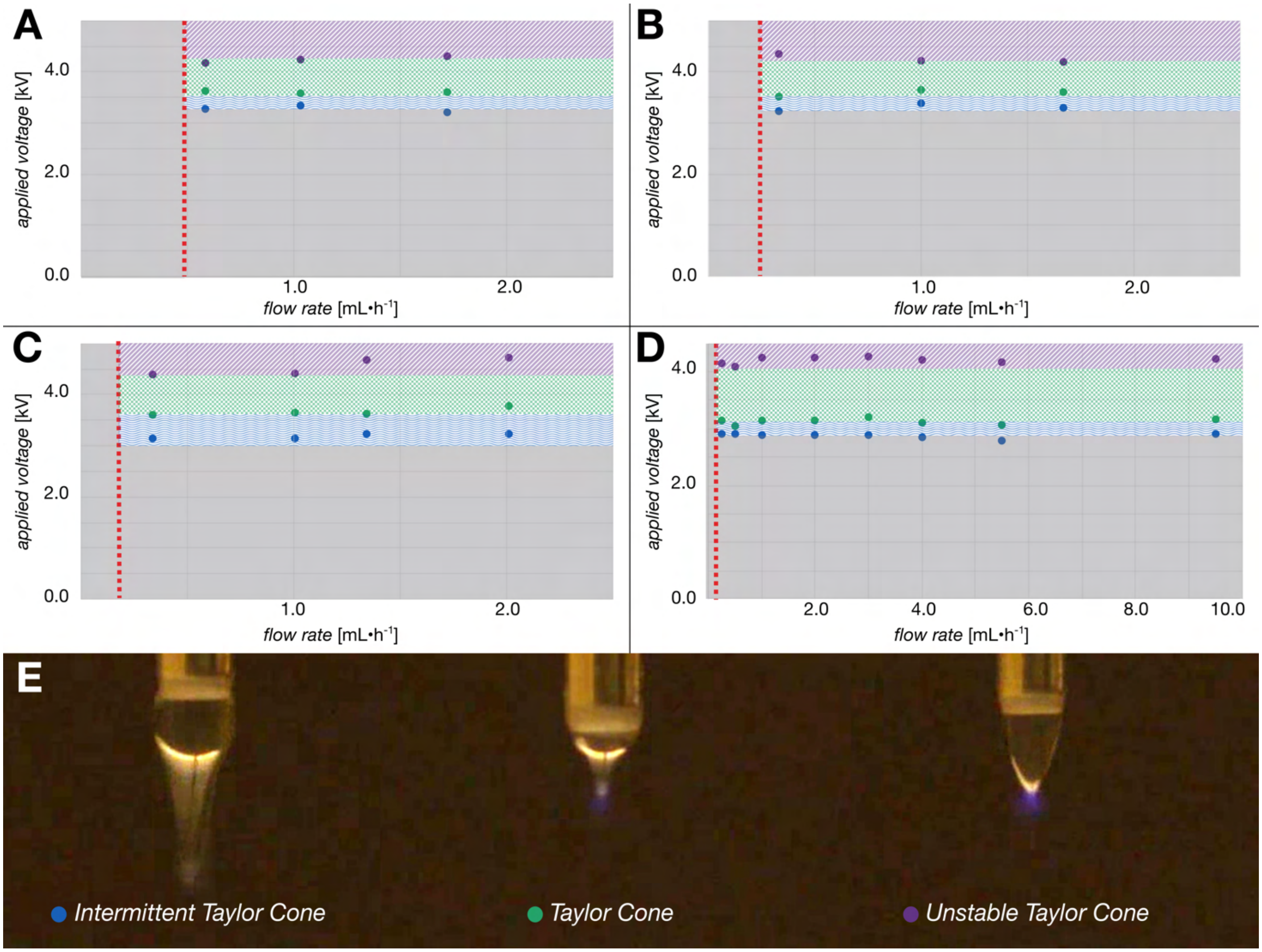

Fig. S2 Voltage–flow rate phase diagrams for electrospraying DEX solutions. Dots indicate reference points for the transition between different modes. The regimes were simplified to linear trends and have been drawn according to the dots: no Taylor Cone (TC) (gray), intermittent TC (blue), stable TC (green), and unstable TC (purple). The red, dotted line indicates the minimal flow rate required to obtain a Taylor cone. Different plots show the TC behavior in case of 5 wt% 100 kDa DEX solution (A), 10 wt% 100 kDa DEX solution (B), 15 wt% 100 kDa DEX solution (C), and 15 wt% 150 kDa DEX solution (D). For A-C, the distance between the needle and ring was maintained at 0.5 cm, while it was 0.2 cm for (D). (E) Representative images corresponding to the three different general TC states, obtained with a 21-gauge needle. It is particularly noticeable in the stable and unstable TC regimes that a prominent corona discharge is observable, though it is considerably more prominent in the unstable TC situation.

B Dynamic Light Scattering Characterization of Vesicles

To characterize the size of the produced vesicles, we performed dynamic light scattering measurements with a benchtop Malvern Zetasizer. The results of the size distribution are presented in Figure S3.

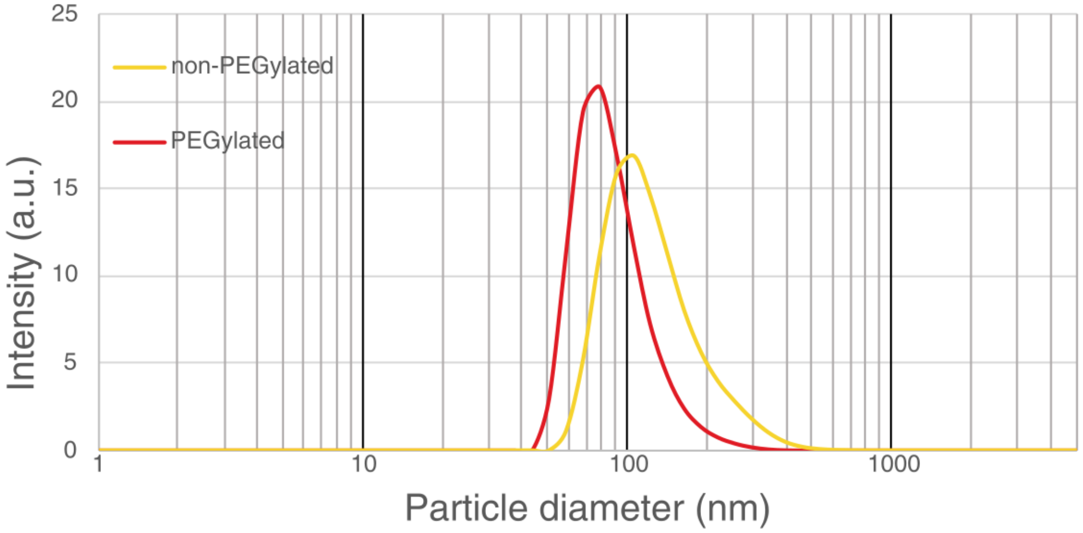

Fig. S3 Size distribution of SUVs prepared via extrusion through polycarbonate membranes with 100 nm pores. Lipid compositions (molar ratios) were as follows: DOPC : DOPG : DOPS : DOPE-mPEG : Rhod-PE, 36 : 36 : 25 : 2.9 : 0.1 for PEGylated SUVs; DOPC : DOPG : DOPS : Rhod-PE, 37.5 : 37.4 : 25 : 0.1 for non-PEGylated SUVs. Vesicle size did not differ considerably between the two populations, with maxima at 80 and 105 nm, respectively.

C Materials

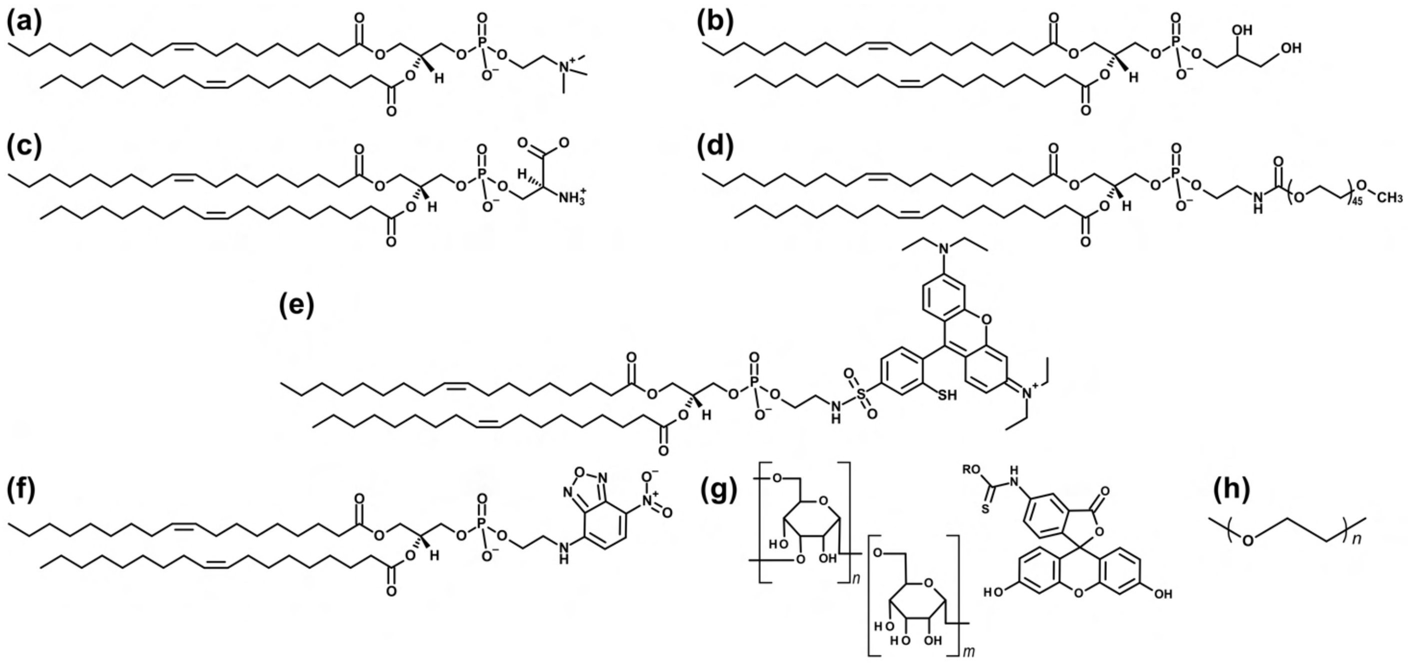

Fig. S4 The chemical structures of the primary molecules used in this study. (a) 1,2-dioleoyl-*sn*-glycero-3-phosphocholine (DOPC); (b) 1,2-dioleoyl-*sn*-glycero-3-phosphoglycerol (DOPG); (c) 1,2-dioleoyl-*sn*-glycero-3-phospho-L-serine (DOPS); (d) 1,2-dioleoyl-*sn*-glycero-3-phosphoethanolamine-N-[methoxy(poly(ethylene glycol))-2000] (DOPE-mPEG 2000); (e) 1,2-dioleoyl-*sn*-glycero-3-phosphoethanolamine-N-(lissamine rhodamine B sulfonyl) (Rh-DOPE); (f) 1,2-dioleoyl-*sn*-glycero-3-phosphoethanolamine-N-(7-nitro-2-1,3-benzoxadiazol-4-yl) (NBD-DOPE); (g) Dextran (from *Leuconostoc supp.*, DEX), both with and without a fluorescein 5-isothiocyanate (FITC) label (shown on the right); (h) poly(ethylene glycol) (PEG).

D Parameters and dimensionless flow numbers associated with our system

To get a better sense of the interplay of physical parameters at play in our electrospray system regarding droplet collection and penetration into the continuous phase, we can look at a set of dimensionless fluid dynamics numbers to estimate the impacts of the different physical parameters. In our cases, the most relevant parameters are the consideration of the effects of interfacial tension, viscosity, and gravity: we thus consider the capillary number Ca, the Weber number We, the Reynolds number Re, and the Bond/Eötvös number Bo. Estimations of the physical parameters relevant to our system in calculating these numbers are presented in Table S1.

Table S1 Estimations of the physical parameters in electrospraying a solution of DEX into PEG.

Symbol	Meaning	Value	Ref.
ρ_{DEX}	density of the DEX solution	$\approx$1080 kg·m^{-3}	1
ρ_{PEG}	density of the PEG solution	$\approx$1020 kg·m^{-3}	1,2
ρ_{air}	density of air	1.22 kg·m^{-3}	
γ_{DEX}	surface tension of the DEX solution	$\approx$70 mN·m^{-1}	3
γ_{ATPS}	interfacial tension between DEX and PEG solutions	$\approx$0.3 mN·m^{-1}	4
η_{air}	air viscosity	1.8×10^{-5} kg·m^{-1}·s^{-1}	
η_{PEG}	PEG solution viscosity	$\approx 9\times10^{-3}$ kg·m^{-1}·s^{-1}	2,5
η_{DEX}	DEX solution viscosity	$\approx 10^{-1}$ kg·m^{-1}·s^{-1}	6
R	typical electrosprayed droplet radius	5×10^{-5} m	
g	local gravitational acceleration	9.8 m·s^{-2}	

The dimensionless flow numbers themselves are presented in Table S2 for our system of dextran droplets being sprayed through air into the PEG solution. To determine velocity v of the falling droplets, we assume that our droplets are accelerated to their terminal velocity in air before they impact the PEG solution, and we estimate the terminal velocity assuming Stokes flow in a low Reynolds number regime:

$$v_{\mathrm{ter}} = \frac{2R^2 g\Delta\rho}{9\eta_{\mathrm{air}}} = \frac{2R^2 g(\rho_{\mathrm{DEX}}-\rho_{\mathrm{air}})}{9\eta_{\mathrm{air}}} \sim 0.33\ \mathrm{m\cdot s^{-1}}$$

We calculate the corresponding Reynolds number:

$$Re = \frac{\rho_{\mathrm{air}} v_{\mathrm{ter}} R}{\eta_{\mathrm{air}}} \sim 1.1$$

While the obtained value does not strictly satisfy the low Reynolds number regime ($Re \ll 1$), it is a reasonable approximation and we treat the obtained numbers only as a rough estimate. Within the ATPS system, we can further calculate the sedimentation velocity of the vesicles in a similar manner:

$$v_{\mathrm{ATPS}} = \frac{2R^2 g(\rho_{\mathrm{DEX}}-\rho_{\mathrm{PEG}})}{9\eta_{\mathrm{PEG}}} \sim 3.6\cdot10^{-5}\ \mathrm{m\cdot s^{-1}}$$

Table S2 Estimates for the dimensionless numbers used in our electrospray set-up for DEX phase droplets landing in the PEG phase. Order of magnitude of the dimensionless numbers aligns with observations of the droplets staying afloat right after impact.

Number	Formula	Meaning	Order-of-magnitude estimate	Remark
Reynolds number (Re_{ATPS})	$\frac{\rho_{\mathrm{PEG}} v_{\mathrm{ATPS}} R}{\eta_{\mathrm{PEG}}}$	$\frac{\text{inertia}}{\text{viscosity}}$	10^{-4}	viscous forces dominant
Capillary number (Ca)	$\frac{\eta_{\mathrm{PEG}} v_{\mathrm{ATPS}}}{\gamma_{\mathrm{ATPS}}}$	$\frac{\text{viscosity}}{\text{interfacial tension}}$	10^{-3}	interfacial tension dominant
Weber number (We)	$\frac{\rho_{\mathrm{DEX}} v_{\mathrm{ATPS}}^2 R}{\gamma_{\mathrm{ATPS}}}$	$\frac{\text{inertia}}{\text{interfacial tension}}$	10^{-7}	interfacial tension dominant
Bond number (Bo)	$\frac{g\Delta\rho R^2}{\gamma_{\mathrm{ATPS}}}$	$\frac{\text{gravity}}{\text{interfacial tension}}$	10^{-2}	droplets prefer to float

These numbers help us conclude that gravitational effects on the droplets are negligible, at least in the initial moments upon contact, and that interfacial tension forces will initially pin the droplets at the interface. This is congruent with our observation that the droplets indeed prefer to float upon impact and only begin sinking with time, if at all, and with the observed slow rate of sinking.

E Additional Images

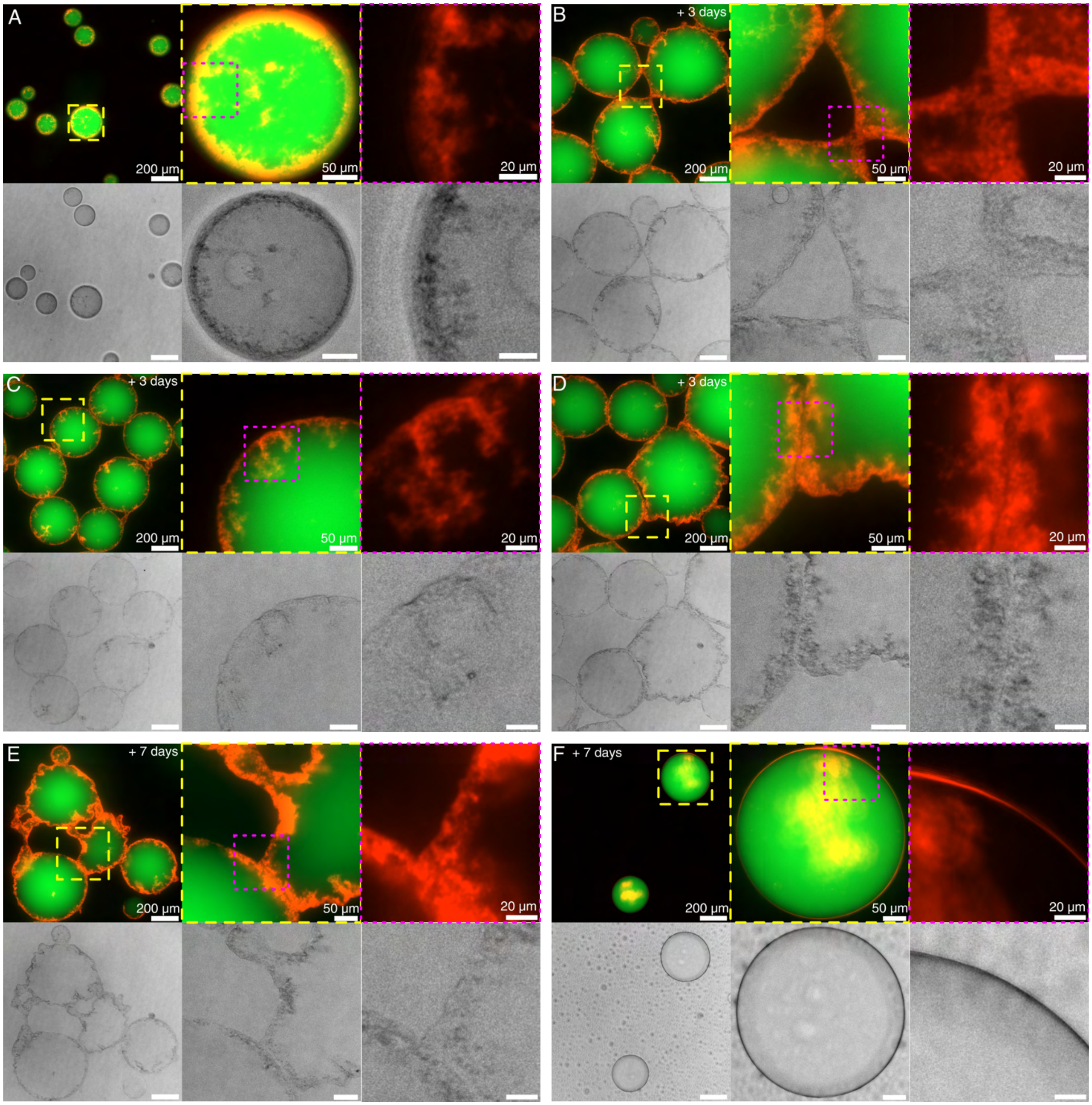

Fig. S5 Detailed close-ups (fluorescence and bright-field) of the lipid structures at the PEG–DEX interface over time. (A) The starting point of the assemblies, with fuzzy lipid shells at the border of the DEX droplets. (B-C) Over time, the lipid structures start interacting with each other, deforming the interface (B) and thus leading to inward and outward protrusions (C) and pronounced lipid–lipid contact sites (D). (E-F) After seven days, some loose droplets have sunk, leaving deformed floating structures with extensive lipid shells (E) and smooth lipid vesicles at the bottom (F). Fluorescence: FITC-DEX (green), Rh-DOPE (red).

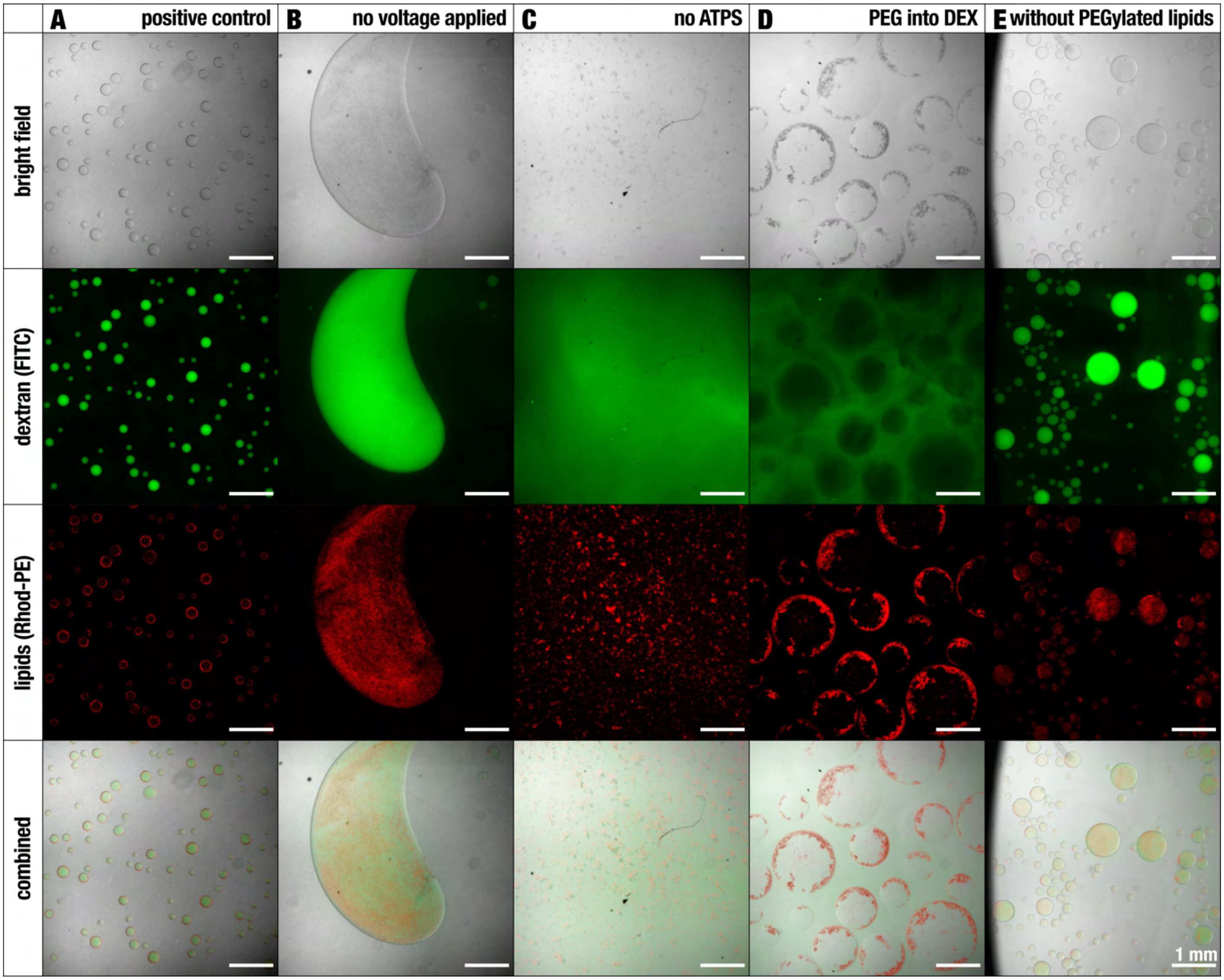

Fig. S6 PEGylation and ATPS are key factors for interfacial lipid assembly. A) Positive control: Electrospraying the DEX-rich phase (containing SUVs with PEGylated lipids) into the PEG-rich phase results in vesicles with lipid nebulae. B) No electrospray: In the absence of an applied electric field, no droplets are formed but a single SUV-laden DEX phase wets the bottom of the dish. C) No ATPS: Spraying the SUV-laden DEX-rich phase into a PBS buffer without PEG produces a suspension of lipid aggregates without any sequestration of DEX. D) Inverted system: By electrospraying a PEG-rich phase (containing SUVs with PEGylated lipids) into a DEX-rich phase, we observe both the sequestration of the PEG phase and the aggregation of lipids at the interface, though with much larger droplets compared to (A), likely due to the lack of optimization towards spraying PEG solutions. E) No PEGylated lipids: The omission of PEGylated lipids in the prepared SUVs severely hampers the interfacial assembly and does not lead to nebulae-like structures, and the lipids remain sequestered inside the droplets along with the DEX. Fluorescence: FITC-DEX (green), Rh-DOPE (red).

References

1 C. Han, S. Takayama and J. Park, *Scientific Reports*, 2015, **5**, 11891.

2 P. Gonzalez-Tello, F. Camacho and G. Blazquez, *Journal of Chemical & Engineering Data*, 1994, **39**, 611–614.

3 M. Hoorfar, M. A. Kurz, Z. Policova, M. L. Hair and A. W. Neumann, *Langmuir*, 2006, **22**, 52–56.

4 D. Forciniti, C. K. Hall and M. R. Kula, *Journal of Biotechnology*, 1990, **16**, 279–296.

5 Y. Neuzillet, S. Giraud, L. Lagorce, M. Eugene, P. Debre, F. Richard and B. Barrou, *Transplantation Proceedings*, 2006, **38**, 2354–2355.

6 V. Tirtaatmadja, D. E. Dunstan and D. V. Boger, *Journal of Non-Newtonian Fluid Mechanics*, 2001, **97**, 295–301.
